# Vaccinia virus induces endoplasmic reticulum stress and activates unfolded protein responses through the ATF6α transcription factor

**DOI:** 10.1186/s12985-023-02122-y

**Published:** 2023-07-11

**Authors:** Thiago Lima Leão, Karine Lima Lourenço, Cid de Oliveira Queiroz, Ângela Vieira Serufo, Aristóbolo Mendes da Silva, Edel F. Barbosa-Stancioli, Flávio Guimarães da Fonseca

**Affiliations:** 1 Departamento de Microbiologia, Instituto de Ciências Biológicas, Universidade Federal de Minas Gerais, Belo Horizonte, Minas Gerais 31270-901 Brazil; 2 Departamento de Morfologia, Instituto de Ciências Biológicas, Universidade Federal de Minas Gerais, Belo Horizonte, Minas Gerais Brazil

**Keywords:** Vaccinia virus, Cell stress, Endoplasmic reticulum stress, Unfolded protein response, MVA, WR

## Abstract

**Background:**

Cell responses to different stress inducers are efficient mechanisms that prevent and fight the accumulation of harmful macromolecules in the cells and also reinforce the defenses of the host against pathogens. *Vaccinia virus* (VACV) is an enveloped, DNA virus, belonging to the Poxviridae family. Members of this family have evolved numerous strategies to manipulate host responses to stress controlling cell survival and enhancing their replicative success. In this study, we investigated the activation of the response signaling to malformed proteins (UPR) by the VACV virulent strain—Western Reserve (WR)—or the non-virulent strain—Modified Vaccinia Ankara (MVA).

**Methods:**

Through RT-PCR RFLP and qPCR assays, we detected negative regulation of XBP1 mRNA processing in VACV-infected cells. On the other hand, through assays of reporter genes for the ATF6 component, we observed its translocation to the nucleus of infected cells and a robust increase in its transcriptional activity, which seems to be important for virus replication. WR strain single-cycle viral multiplication curves in ATF6α-knockout MEFs showed reduced viral yield.

**Results:**

We observed that VACV WR and MVA strains modulate the UPR pathway, triggering the expression of endoplasmic reticulum chaperones through ATF6α signaling while preventing IRE1α-XBP1 activation.

**Conclusions:**

The ATF6α sensor is robustly activated during infection while the IRE1α-XBP1 branch is down-regulated.

## Background

The *Vaccinia virus* (VACV), the prototype of the *Poxviridae* family, is a large double-stranded DNA virus with a brick-shaped enveloped particle and a genome that ranges from 178 to almost 200 kb, depending on the virus’ strain, which codes for approximately 200 ORFs [1–3]. Up to 50% of the virus' genome codes for immune evasion-related and/or host-interaction genes, a feature shared by many other poxviruses. Western Reserve (WR) is a virulent VACV strain originally derived from New York City Board of Health (NYCBH) virus—a vaccine strain used during the smallpox eradication campaign [4, 5]. Multiple passages of the NYCBH virus in diverse cell types and animals like rabbits and mice induced an array of mutations in the virus genome, making the resulting WR strain considerably pathogenic to a wide range of mammals and unsuitable to be used as vaccine against smallpox [2, 6]. Nonetheless, the WR strain became a model poxvirus commonly utilized in most studies looking at the biology of poxviruses. The Modified virus Ankara (MVA) strain, on the other hand, is an attenuated strain that is avirulent for humans and other mammals as the virus is unable to fully replicate in most mammalian cells [7–9]. MVA was obtained after more than 570 passages of its parental strain in primary chicken embryo fibroblast cells (CEFs), culminating in the development of an immunogenic and highly attenuated strain. As a vaccine against smallpox, clinically tested, the virus was well tolerated by vaccinees and demonstrated that it can be used to confer cross-protection against Variola virus (the etiological agent of smallpox) [10]. Complete genome sequence analysis of MVA revealed six major deletion sites on its genome as well as numerous point mutations—a consequence of the cell passaging—encompassing a total reduction of 31 kb on its coding capacity when compared to the parental virus, the Chorioallatoid Ankara strain [11].

All members of *Poxviridae* family replicate entirely in the cytoplasm of the host cell and depend largely on viral proteins for their replication; however, these complex viruses also exploit numerous cellular pathways to ensure their replicative success. The VACV replication occurs in close association with the endoplasmic reticulum (ER) [12], the largest membranous organelle of most eukaryotic cells, where most nascent proteins are folded. Additionally, the ER is essential for the balance of the intracellular calcium and the organelle plays a key role in the lipids and sterols biosynthesis [13]. Due to its participation in many important cell processes, the ER is sensitive to perturbations in the cellular homeostasis triggered by stresses from endogenous or exogenous origins. Sources of perturbation includes chemical damage, genetic mutations, nutritional starvation, cell differentiation and, of course, infection by different intracellular pathogens [14–18]. The resultant disturbances in cell homeostasis can alter nascent protein formation, leading to the accumulation of unfolded or misfolded proteins inside the ER, a condition known as ER stress. This condition triggers ER stress responses, which are generally known as unfolded protein responses (UPR).

There are three major signaling pathways that are part of the UPR. These pathways are controlled by the following ER-resident sensor proteins: the inositol-requiring protein 1 alpha (IRE1α); the activating transcription factor 6 alpha (ATF6α) and the protein kinase RNA-like ER kinase (PERK). Together, these pathways are responsible for surveillance of the ER stress. Depending on the cell type and stimuli as well as of the duration of the stress condition, the outcome of these signals may result either in the recovery of protein homeostasis or cell death [19–21]. These sensors are able to attenuate protein translation but also increase the expression of ER chaperones and ERAD components which, in turn, culminate in the increment of the ER capacity and/or reduction of the ER demand, restoring the organelle homeostasis.

Given that endoplasmic reticulum is an organelle exploited by VACV for replication, we examined the ER homeostasis during the virus infection. Here, we describe how virulent or attenuated strains of VACV can affect the UPR signaling pathway and the importance of UPR components for virus multiplication in murine embryo fibroblast cells.

## Materials and methods

### Cells, viruses and infection conditions

Primary chicken embryo fibroblasts (CEF) were prepared as described [22, 23]. BSC40 (ATCC CRL-2761), BALB/3T3 clone A31 (ATCC CCL-163), PERK-WT (formerly known as DR-Wildtype, ATCC CRL-2977), PERK-KO-DR (ATCC CRL-2976), immortalized ATF6 knockout mouse embryonic fibroblasts (MEFs) and ATF6-wildtype control cell—a kind gift of Dr. Randall Kaufman [24]—were grown under standard conditions in Dulbecco's modified Eagle's medium (DMEM) supplemented with 2 mM L-glutamine, 0.1 mM Non-Essential Amino Acids (NEAA), 10% heat-inactivated fetal bovine serum, 100 units/ml penicillin, and 100 µg/ml streptomycin in a humidified atmosphere with 5% CO_2_ at 37 °C.

*Vaccinia Virus* strains Western Reserve (WR) and Modified Vaccinia Ankara (MVA) were gifts from Dr. Bernard Moss (NIH, Bethesda). Viruses’ stocks were propagated in CEF and BSC40 cells for MVA and WR, respectively, and purified in sucrose cushions as described [25]. Viral titers were determined by plaque assay for WR or immunostaining for MVA [23, 26].

All VACV infections in MEF cells were performed at the multiplicity of infection 10 (unless otherwise indicated) in the absence of FBS for 1 h and cultured in DMEM 2.5% FBS after which unabsorbed viruses were removed. When required, VACV was UV-inactivated after exposure of virus samples for 20 min to an UV lamp producing irradiation predominantly at 365 ηm. UV-irradiated viruses were tested for infectivity prior to use. Viruses that were no longer capable of forming plaques compared with the non-irradiated samples were assumed to be UV-inactivated. DNA synthesis inhibitor 1-b-D-arabinofuranosylcytosine (AraC) (sigma), when used, was added 30-min before infection period and throughout the infection to a final concentration of 50 µg/ml.

### Characterization of reporter gene expression

On the day before transfection, MEF cells were seeded in 24-well plates (1 × 10^5^ cells/well) in 10% FBS DMEM. Cells were co-transfected in triplicates with 0.2 µg of p5xATF6-GL3 (a generous gift from Dr. Ron Prywes, Department of Molecular Genetics, Kumamoto University, Japan) [27] and 0.05 µg of pRL-TK (Promega), using Lipofectamine 2000 (Invitrogen) accordingly to the protocol provided by the manufacturer. Transfected cells were infected with VACVs 24 h past transfection at MOI 10 or treated with 2.5 µM Tunicamycin (Sigma). At indicated hours post infection (hpi) the growth medium was removed, cells were rinsed with PBS 1 × and lysed by shaking culture flasks for 15 min on ice with 100 μl of Passive Lysis Reagent (Promega). Twenty microliters of each cell lysate were assayed for firefly and renilla luciferase activities using a LumiCount Microplate Reader Luminometer (Packard BioSciences) and Dual-Luciferase Reporter Assay System (Promega) accordingly to the protocols provided by the manufacturers. Results were expressed as the ratio between the activities of firefly luciferase and renilla luciferase (RLA, Relative Luciferase Activity).

### Characterization of nuclear translocation of reporter gene

The day before transfection, A31 cells were plated on coverslips in 24-well plates. Cells were transfected with 0.6 µg of pShortCMV-ATF6-GFP, a kind gift from Kazutoshi Mori (Kyoto University, Japan) [28], as previously described. Cells were, then, infected with VACV or treated with tunicamycin for 24 h, counterstained with Hoechst 33,258 (Sigma) and coverslips were mounted on glass slides using ProLong Diamond Antifade Mountant (Life Technologies). The reporter gene expression and localization were assessed by fluorescent microscopy using EVOS FL cell imaging system (Life Technologies). To quantify nuclear translocation of reporter gene, we isolated the nucleus from transfected and infected cells in ice-cold nuclear isolation buffer (320 mM sucrose, 5 mM MgCl_2_, 10 mM HEPES and 1% Triton X-100 pH 7.4). We further verified nuclear integrity by trypan stain and then counted GFP positive nuclei by flow cytometry in a BD FACScan cytometer.

### RNA extraction and cDNA synthesis

Total RNA was extracted from cells using TRI Reagent (Sigma) according to the manufacturer’s instructions and treated with rDNase I (Macherey–Nagel) before reverse transcription to remove residual genomic DNA contamination. Extracted RNA was dissolved in diethypyrocarbonate (DEPC)-treated water, and the RNA concentration and purity were estimated on a NanoVue Plus Spectrophotometer (GE Healthcare Life Sciences). Aliquots of RNA samples were subjected to electrophoresis in a 1% denaturing agarose gel containing ethidium-bromide staining to verify RNA integrity. The cDNA was synthesized from 1 μg RNA in a final reaction volume of 20 μl, using Moloney murine leukemia virus (M-MLV) reverse transcriptase and random primers (Promega) according to the manufacturer's protocol. The reverse transcription product was stored at − 20 °C until use.

### Measurement of XBP1 splicing by agarose gel electrophoresis

One tenth of cDNA’s volume was used per PCR reaction in a 25-μl reaction volume using GoTaq^®^ Flexi DNA Polymerase (Promega) according to the manufacturer's instructions. The cycling conditions were 94 °C for 4 min, followed by 30 cycles of 94 °C for 30 s, 68 °C for 30 s, and 72 °C for 30 s. For detection of XBP1 isoforms (spliced and unspliced), the sense primer mXBP1.3S (5ʹ-AAACAGAGTAGCAGCGCAGACTGC-3ʹ) and the antisense primer mXBP1.2AS: (5ʹ-GGATCTCTAAAACTAGAGGCTTGGTG-3ʹ) were used to amplify a 600-bp cDNA product encompassing the IRE1α cleavage sites [29]. This amplified fragment was further digested with PstI (Promega) to reveal a restriction site that is lost after splicing of the XBP1 mRNA. The amplicon fragments were resolved on a 1.4% agarose gel containing ethidium-bromide (Life Technologies). Gels were imaged using a UVP MultiDoc-It Imaging System. Bands were quantified using ImageJ (NIH).

### Quantitative real-time PCR

One 100-fold diluted cDNA was used to determine the levels of BiP and PDIA4 mRNA by quantitative PCR, using SYBR Green Master Mix (Applied Biosystem) and water in10 μL final volume reactions. Relative gene expression analyses were performed using the 2^−ΔCt^ method and normalized to the expression of RPL32, HPRT and GAPDH mRNA [30]. Primers used for the qPCR were described by Mügge and da Silva [31].

### Viral infectivity assays

Knockout cells and respective wild type control cells were grown to a density of 1 × 10^6^ cells per well on 6-well culture dishes and then infected with VACV-WR. Infections were carried out at a MOI of 10 for 3, 6, 12, 24 and 48 h. The infected monolayers were then harvested and titrated in BSC-40 cells for one-step growth curve assays. For Multi-step growth analysis, 7 × 10^5^ cells were infected with MOI 0.01 and harvested at 0 (at the end of adsorption), 24, 48 and 72 h. Virus titers were determined as described before. VACV-WR plaque phenotype assays were carried in cells infected at 0.01 MOI and incubated for 48 h. For transmission electron microscopy, ATF6-WT and ATF6-KO cells were plated on 35 mm diameter plates and infected with VACV WR at a multiplicity of infection of 10. After 24 hpi the cells were mechanically dislodged, centrifuged for 5 min at 1500 × rpm and washed 2 × with 1X PBS. After washing, Cells were fixed by shaking gently for 1 h in 2% gluteraldehyde-0.1 M sodium cacodylate buffer and, then, washed twice with 0.1 M cacodylate buffer. After washing, cells were post-fixed with 2% osmium tetroxide buffer and embedded in EPON resin. Samples were ultra-sectioned and the sections were analyzed and photographed in a Tecnai G2-12—SpiritBiotwin FEI-120 kV transmission electron microscope. The Genetic complementation assay was performed by rescuing ATF6 expression in knockout cells. In this experiment, ATF6 gene was reintroduced by transfection of pEGFP-ATF6 (Addgene plasmid # 32,955) [32] which contain a strong promoter controlling the heterologous expression. Control cells were transfected with the plasmid pEGFP-C1 (empty vector) (Clontech Laboratories). Total DNA was extracted using UltraPure Phenol:Chloroform:Isoamyl Alcohol (25:24:1) (Invitrogen) and viral genome quantitation was performed by qPCR using a primer set to amplify the CEV DNA sequence [33] and comparing to a standard curve from purified VACV genomes.

## Results

### Activation of ATF6α during VACV infection

**Fig. 1:**
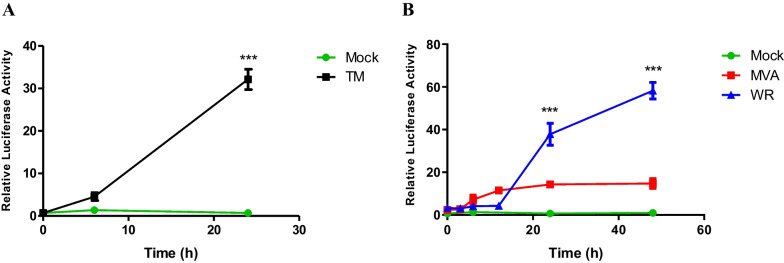
5 × ATF6 reporter activation kinetics over the course of VACV infection in MEFs. **A** A31 MEFs were transfected with p5xATF6-GL3 and pRL-TK. and treated with 3 µM of tunicamycin for 0, 6 and 24 h. Cell lysates were collected and assayed for Firefly and Renilla luciferase activities. **B** Transfected A31 cells were infected with WR or MVA (MOI 10) and harvested at different times points post infection. Asterisks indicate statistically significant differences (*p *< 0.001) between groups as determined by two-way ANOVA followed by Bonferroni’s test. The data was plotted as means ± SEM of duplicate samples from three independent experiments

To determine whether VACV infection can activate ATF6 transcriptional activity we carried out ATF6α reporter gene assays in A31 MEFs. Cells were transfected with the p5xATF6-GL3 luciferase expression plasmid in which the luciferase gene is controlled by five repeats of the ATF6α DNA binding site (TGACGTG) [27]. First, we determined that uninfected A31 MEFs (mock control) are responsive when treated with tunicamycin, a glycosylation inhibitor and ER stress inducer agent (Fig. 1a). To evaluate the virus effects on ATF6α activation, cells were transfected with the luciferase expression plasmid and further infected with VACV WR or MVA. Cell lysates were harvested at 0, 3, 6, 12, 24 and 48 h post infection (hpi) and we detected that both virus’ strains can induce noticeable increase in ATF6α reporter activity during the course of infection (Fig. 1b), with distinguishable intensities. Viruses are able to induce ATF6α transcriptional activity three hours after adsorption but this is significantly different from mock-infected cells only after 12 hpi and 24hpi, for MVA and WR strains, respectively (Fig. 1b). Once initiated, WR-induced ATF6α transcriptional activity is significantly higher than that observed for MVA-induced activity, either 24 or 48 hpi. (Fig. 1b).

### ATF6α is translocated to the nucleus after VACV infection

**Fig. 2:**
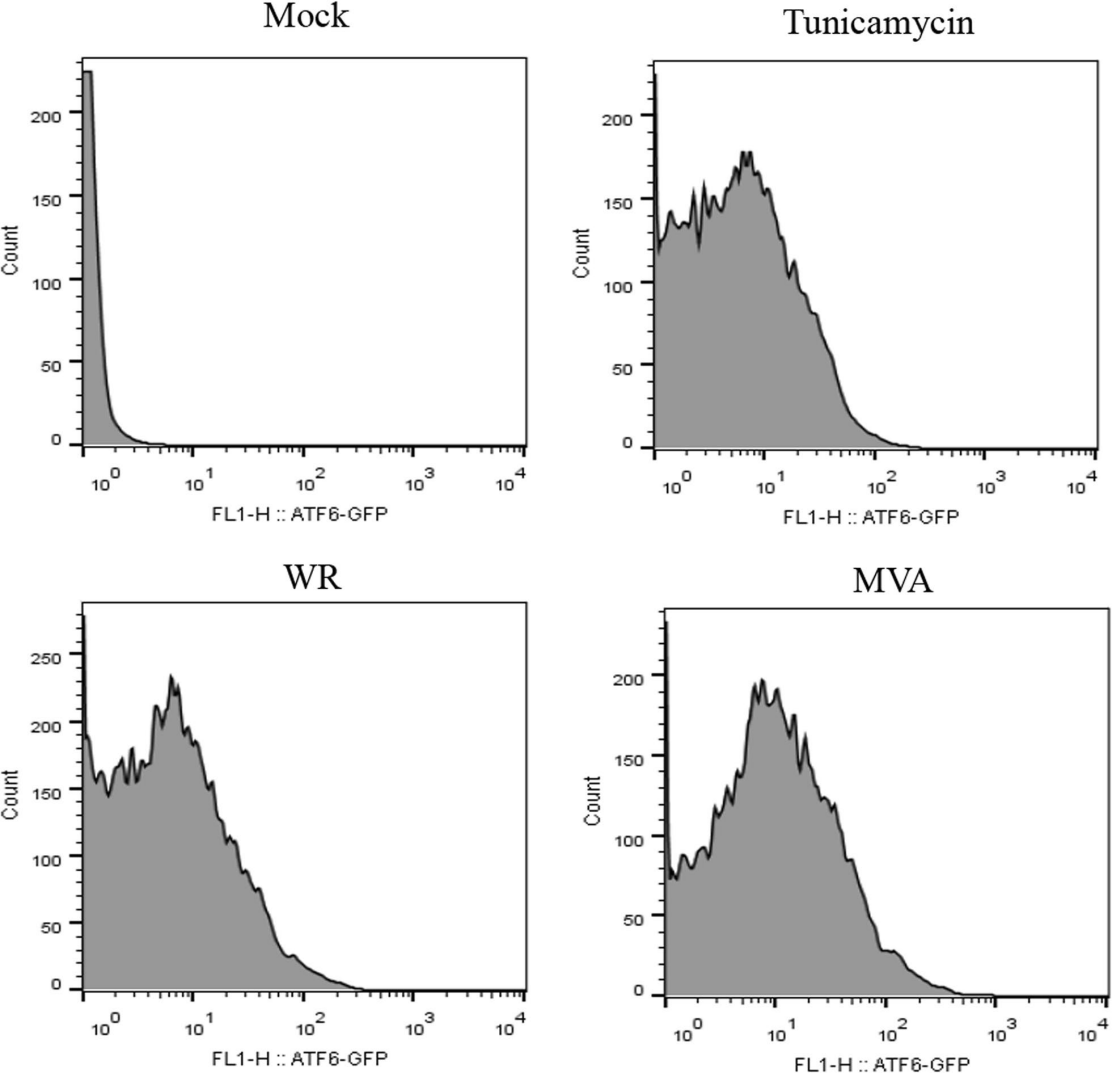
ATF6-GFP reporter nuclear translocation in VACV infected MEFs. A31 MEFs were transfected with the pCMVshort-EGFP-ATF6α and infected with VACV strains for 24 h. Cells were carefully collected and lysed with nuclear isolation buffer. Isolated nuclei obtained after washing were analyzed for green fluorescence in a FACSCan flow cytometer. 10.000 events were collected inside the gate determined for nuclei size and granulosity. Results were plotted as histograms for green fluorescence intensity versus cell counts

An alternative approach to evaluate ATF6α activation and effects on transcriptional activity is to evaluate its nuclear translocation upon the stress event. Therefore, we performed experiments using eGFP (enhanced green fluorescent protein)-ATF6α fusion protein which is localized in the ER under unstressed conditions, and then is translocated to the nucleus upon activation, similarly to the endogenous ATF6α [28]. A31 cells were transfected with pShortCMV-ATF6-GFP, infected with VACV WR or MVA and reporter gene expression and localization was assessed by fluorescent microscopy. Furthermore, nuclei were isolated and analyzed by flow cytometry. As noted in Fig. 2, there was an increase in the percentage of GFP-ATF6-positive nuclei in infected cells when compared to uninfected ones. Such increase was comparable to cells treated with tunicamycin.

### VACV late gene expression is required for maximum ATF6α transcriptional activity

**Fig. 3:**
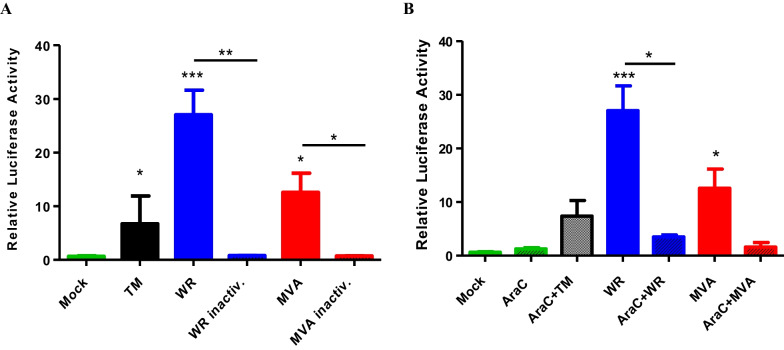
5 × ATF6 reporter activation is dependent on viral biosynthesis in MEFs. **A** A31 MEFs were transfected with p5xATF6-GL3 and pRL-TK. Cells were exposed to UV-inactivated virus or incubated with arabinose C (AraC) (50 µg/ml) for 30 min prior to virus infection. Incubation was carried in the presence of the inhibitor. Cell lysates were collected and assayed for Firefly and Renilla luciferase activities. Tunicamycin-treated (1 µM) cells lysates were used as positive control. The data are mean of duplicate samples from two independent experiments. *, ** and *** indicate statistically significant differences in relation to untreated cells (*p *< 0.05; *p *< 0.01; *p *< 0.001 respectively) as determined by unpaired two-tailed Student’s T-test

Next, we investigated whether activation of the 5 × ATF6α reporter was dependent on viral biosynthesis. In order to look into that we inoculated cells with UV-treated VACV-WR and MVA particles, and observed that ATF6α reporter activity was completely abrogated (Fig. 3a). However, when cells were infected with intact virus’ particles and co-treated with the replication inhibitor cytosine arabinoside (AraC)—an nucleotide analog that allows complete early viral gene expression but repress intermediate and late gene expression as well as the virus replication, a significant decrease in the ATF6α reporter activity was observed, but not complete abrogation (Fig. 3b). These results indicate that viral infection is necessary for ATF6α activity using the reporter assay.

### IRE1α-dependent splicing of X-box binding protein 1 (XBP1) mRNA remains at basal levels during VACV infection

**Fig. 4:**
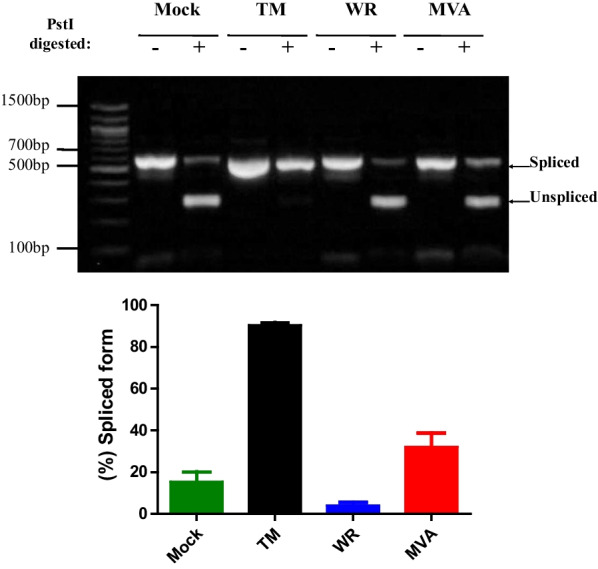
XBP1 mRNA splicing in VACV infected A31 MEFs. **A** A31 cells were infected with VACV at a MOI of 10 or treated with tunicamycin (TM) for 24 h. Total RNA was extracted and XBP1 mRNA transcripts were amplified by RT-PCR, digested with PstI, and DNA fragments were resolved on an ethidium bromide-stained agarose gel (upper panel). The spliced transcript lacks a PstI site and migrates slower than the digested unspliced transcript. Densitometry analysis of XBP1 spliced isoform was plotted as bar diagrams showing means ± SEM of three replicates (bottom panel). *, ** and *** indicate statistically significant differences between samples (*p *< 0.05; *p *< 0.01; *p *< 0.001 respectively) as determined by one-way ANOVA followed by Bonferroni’s test

During the ER stress, IRE1α exhibits endonuclease activity by removing a residual 26 nt intron from the XBP1 transcription factor mRNA. XBP1 is a basic leucine zipper (bZIP)-containing transcription factor of the CREB/ATF protein family that binds to the UPR element (UPRE) upstream of responsive genes. Previous studies have demonstrated that basal or tunicamycin-induction of 5 × ATF6α reporter may require, in some instances, the XBP1 spliced isoform [24]. Using a RT-PCR RFLP assay to monitor XBP1 splicing with specific primers and also exploiting a restriction enzyme *PstI* site present within the residual intron to distinguish between the unspliced and spliced forms of the XBP1 transcript, we detected that the VACV infection do not increase XBP1 mRNA splicing (Fig. 4).

### VACV infection triggers ATF6α-dependent expression of BiP/Grp78 chaperone

**Fig. 5:**
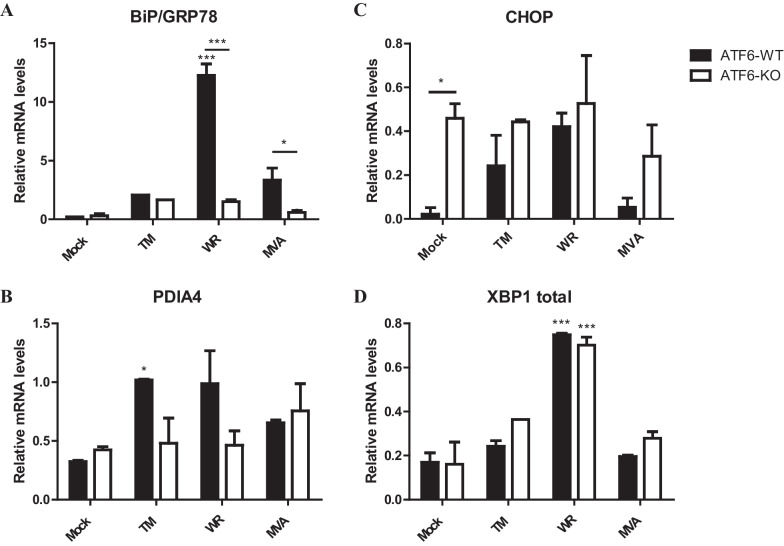
Regulation of ER stress target genes in wild-type MEFs or ATF6α deficient cells. Wild-type and ATF6α knockout MEFs were infected with MOI 10 or treated with 1 µg/mL tunicamycin. Total RNA was extracted and RT-qPCR analysis of relative mRNA contents for BiP/GRP78 (**a**), PDIA4 (**b**), CHOP (**c**), and total XBP-1 (**d**) were performed after cDNA synthesis. * or *** indicate statistical significant differences between groups (*p *< 0.05 or *p *< 0.001, respectively) as determined by ANOVA followed by Bonferroni’s test. Data were collected from three independent experiments. Results were plotted as mean ± SD

The ATF6α transcription factor upregulates a large number of genes involved in organelle homeostasis, such as the ER chaperones. To determine whether VACV infection induces the transcription of UPR target genes in response to ATF6α activation, we used quantitative PCR (qPCR) to monitor the mRNA levels of the immunoglobulin binding protein (BiP), a major chaperone of the ER that directly responds to ER stress and also interacts with all sensors of the UPR signaling chain. We observed that BiP mRNA was strongly induced in tunicamycin-treated cells and particularly in VACV infected-cells (Fig. 5a) when compared to mock treated samples; however, such induction was significant lower in infected cells lacking ATF6α. Cells infected with VACV-WR have relatively high mRNA levels when compared to mock cells in an ATF6-dependent manner. However, the MVA showed no dependence of ATF6 for induction of PDIA4 or p58IPK (Fig. 5b). Ddit3, a pleiotropic gene encoding for CHOP transcription factor, is also a target of the UPR and is commonly associated with apoptosis. We detected increased mRNA transcription levels either after VACV-WR infection or tunicamycin treatment in wild type MEFs. In this case, however, MVA infection did not cause noticeable increments on CHOP mRNA levels when compared to non-infected MEFs. Interestingly, in the absence of ATF6α, the mRNA levels of CHOP were up regulated in cells under different conditions, including non-infected or MVA-infected ones (Fig. 5c). In addition, transcription of the *Xbp1* gene is significantly induced during VACV-WR infection in relation to mock-treated cells, but not during MVA infection or in tunicamycin treated cells. Nonetheless, the increment in the XBP1 mRNA levels was apparently independent of the ATF6α sensor during VACV-WR infections (Fig. 5d).

### Signaling downstream to ATF6α mediates VACV replication in MEFs

**Fig. 6:**
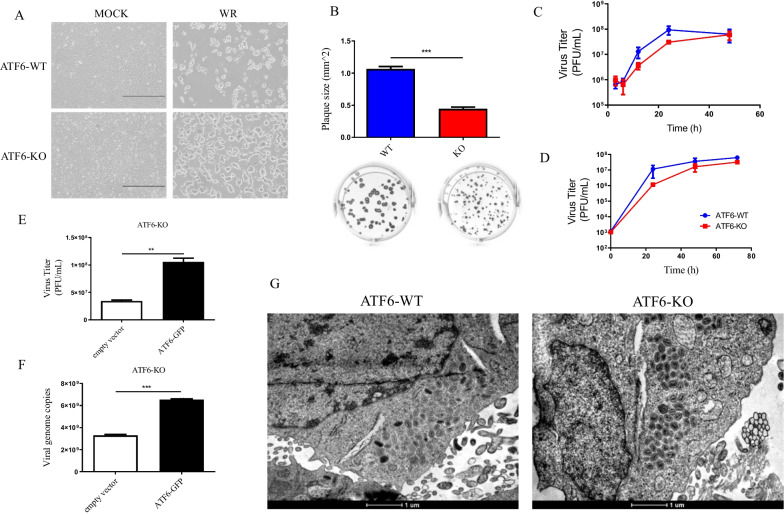
–VACV gene expression, cytopathic effects and viral replication in ATF6α-KO MEFs. **A** Representative Phase-Contrast micrographs of VACV-induced CPE on confluent monolayers of ATF6α-KO or WT MEFs. Cells were infected with MOI 10 and visualized at 24 h post infection. Scale bars indicate 100 µm. **B** ATF6-KO or ATF6-WT MEFs were infected at MOI 0.01 of VACV WR strain for 48 h. Plaque phenotypes were visualized by immunostaining with VACV hyperimmune serum followed by protein-A-HRP and 3, 3′-diaminobenzidine incubation. Representative images are shown. The areas of 50 plaques per infected cell type were measured using ImageJ, and the average ± standard deviation is graphically indicated above each image. **C** Cells were infected with VACV at a MOI of 10 and harvested at 3, 6, 12, 24 or 48 hpi; viral yield was quantified by viral plaque assay in BSC40 cells. Data are means of triplicate experiments ± SD. **D** cells were infected with VACV at a MOI of 0.01 and harvested at 0, 24, 48 or 72 hpi, and viral yield was quantified by viral plaque assay in BSC40 cells. Data are representative of at least three independent experiments. **E** ATF6α-KO MEFs were transfected with pEGFP-ATF6 expression plasmid or empty vector and infected with VACV-WR for 24 h before harvested for virus growth analysis. Virus titers were determined by viral plaque assay in BSC40 cells. Results were plotted as mean ± SEM from duplicate samples and from two independent experiments. **F** Total DNA was extracted from transfected infected-cells and used to quantify VACV genome copy number by qPCR. The data were mean from two independent experiments. ** or *** indicate statistical significant differences between groups (*p *< 0.01 or *p *< 0.001, respectively) as determined by ANOVA followed by Bonferroni’s test. **G** MEFs were infected with VACV WR at MOI of 10, incubated for 24 h, processed and visualized by transmission electron microscopy (TEM). Scale bars are represented bellow each panel

To determine whether VACV-induced UPR signaling and expression of UPR-targeted genes affects the VACV life cycle, we analyzed the cytophatic effect (CPE) and observed that ATF6α-KO MEFs infected with VACV-WR showed remarkably distinct cytophatic effect (CPE) when compared to the infected-WT MEFs (Fig. 6a) as observed in phase contrast micrographs. Likewise, lysis plaque sizes were significantly reduced in ATF6α-KO-infected monolayers when compared to plaque sizes in WT-MEFs infected cells (Fig. 6b). Diameters were 0.444 ± 0.027 mm in ATF6α-KO as compared to 1.066 ± 0.039 mm in ATF6α-WT MEFs. To evaluate virus productivity in the presence or absence of ATF6α, one-step growth curves were produced. ATF6α-KO and wildtype control MEFs were infected with MOI 10 and harvested at 3, 6, 12, 24 and 48 hpi. As showed in (Fig. 6c), the average amount of viable VACV-WR particles at 12 and 24 hpi was apparently lower in ATF6α-knockout cells when compared to wildtype MEFs, although this was not statistically supported. Similar results were obtained in multi-step growth curves using MOI of 0.01 and extending infections for up to 72 h (Fig. 6d). VACV-WR morphogenesis is apparently unaffected by the presence or absence of ATF6 expression, as normal virus particle maturation and accumulation is observed in both conditions (Fig. 6g). On the other hand, using a reverse approach, when ATF6α is transiently overexpressed by transfecting ATF6α-encoding plasmid in cells lacking the endogenous molecule, we observed significant increase (*p *< 0.0045) in the virus yield (Fig. 6e) and viral genome copy numbers (Fig. 6f) at 24 hpi. Analyses of virus replication were carried out for VACV-WR only as MVA does not fully replicate in MEFs.

## Discussion

Cellular stress responses encompass critical mechanisms that prevent cells from accumulating macromolecular damage so that metabolism equilibrium is attained and efficient host defenses against pathogens are mounted. Counteracting such cell strategies, poxviruses have evolved numerous mechanisms to cope with cellular stress responses [34]. In this work, we analyzed the modulation of the UPR signaling during VACV infection and the subtle impact of UPR on VACV in vitro infectivity. To that end, we evaluated the unfolding of UPR during infections by replicative and non-replicative VACV strains—WR and MVA, respectively. Upon activation, the ER stress sensor ATF6α transcription factor undergoes dissociation from BiP, exposing a signal to relocate the sensor to the Golgi network where it is cleaved by S1P and S2P proteases [35] resulting in the release of an amino-terminal fragment. The resultant ATF6α fragment translocates to the nucleus, where it promotes expression of chaperones and genes that code for transcription factors, which play important roles in ER stress, induced apoptosis and proteostasis [36–38]. Our results indicate that VACV infections activate ATF6α-mediated stress-related signaling and that such responses may be beneficial for virus replication.

We used reporter assays to determine nuclear translocation of ATF6α and measure their transcriptional activity upon VACV infection. We detected ER-to-nucleus translocation of ATF6α in either VACV-WR or MVA infected-cells, and this was similar to what was observed in stress induced, tunicamycin-treated positive controls. Likewise, we observed the robust activity of ATF6α-controlled luciferase reporter gene over the course of VACV infections, which was distinguishable from mock-treated cells at late stages of infection. The ATF6α activation upon infection has been observed for other large DNA viruses as well [18, 39, 40]. Although both VACV-WR and MVA are able to induce ATF6α activity, the kinetics and maximum levels of activation are different, suggesting that activation of the UPR-ATF6α branch at early/intermediate stages of viruses’ infection may be driven by WR-encoded genes that are possibly defective or absent in the MVA genome [9]. These results may suggest an impact of viral morphogenesis on the activation of ATF6α. The MVA strain infection produces atypical IMVs in non-susceptible cells and cannot undergo subsequent steps in morphogenesis. The blocking in MVA morphogenesis includes the IMV membrane wrapping, which is known to exploit the Ras-related protein Rab-1A [41]. The puzzle of membranes acquisition by VACV virions has been solved in the last years, shedding light on the crucial role of ER in the VACV life cycle [42–45].

Interestingly, whereas ATF6α was significantly activated during VACV replication, our data suggested that the endoplasmic reticulum (ER) stress-induced activation of IRE1α was attenuated on a specific, crucial point: the IRE1α-mediated XBP1 mRNA splicing was down regulated in VACV-infected cells. During ER stress, IRE1α undergoes dissociation from BiP and BAX inhibitor 1 [46, 47] triggering its dimerization, autophosphorylation and activation of its endonuclease activity [48–50]. The IRE1α nuclease domain has homology to RNase L and its activation causes splicing of a residual intron (26nt) in the XBP1 mRNA, resulting in a more stable and active form of the XBP1 protein. We observed a drop of IRE1α mediated XBP1 mRNA splicing during VACV infection by RT-PCR-RFLP and qPCR (not shown). This VACV infection-associated decrease in XBP1 mRNA splicing is so remarkable that virus infection is able to counteract the tunicamycin-induced XBP1 splicing, considering that the drug is a potent inducer of UPR activation and IRE1α activation. Importantly, it has been demonstrated that increased ATF6α expression results in diminished IRE1α production in human cells, whereas the shut-off of ATF6α causes increased XBP1 mRNA splicing and IRE1α activity, suggesting that IRE1 is controlled by an ATF6-dependent switch off mechanism during ER stress [51]. Our results fit well into this model, as the VACV-induced increase in ATF6α activation correlated with a decrease in XBP1 mRNA processing. Furthermore, the XBP1 transcription factor is essential for sustained cytokine production induced by Toll-like receptors [52] as well other major immune processes [53–56]. Therefore, it seems plausible this virus has adapted evolutionary strategies to down regulate XBP1 mRNA splicing as a way to cope with the host innate responses. Indeed, it has been shown that a VACV protein encoded by the E8L ORF is able to bind to the protein disulfide isomerase-associated 6 (PDIA6) factor, limiting IRE1 activation [57, 58]. However, whether XBP1 production is directly affected by a virus-coded protein or its diminished expression is an indirect function of the increase in ATF6α activity remains to be determined.

One interesting observation that we have made in this work was that UV-inactivated viruses were not capable to stimulate ATF6α transcriptional activity using a luciferase reporter system, suggesting the requirement of VACV biosynthesis to trigger UPR. However, when infections were carried out with functional viruses but in the presence of a viral DNA replication inhibitor (Ara,C), residual ATF6α transcriptional activity was detected. UV treatment of viruses, especially DNA viruses, leads to a block in both transcription and DNA replication [59], whereas AraC blocks VACV DNA replication and abrogates late gene expression [60]. Because we observed continuing ATF6α transcriptional activity in AraC treated cells infected with VACV and no transcription of the luciferase reporter when UV-inactivated viruses were inoculated into cells, we concluded that early viral protein synthesis is able to induce ATF6α activation. Nonetheless, viral DNA replication and subsequent protein synthesis are required for optimal ATF6α activity.

The UPR-targeted genes tested in our study are up regulated during VACV infection in an ATF6α-dependent manner. This conclusion was based upon the fact that molecules such as BiP and PDIA4 had their mRNA levels significantly reduced in ATF6α knockout cells upon infection. Exception was the apoptotic-related CHOP transcription factor, for which mRNA levels increased consistently in the absence of ATF6α. Indeed, Klymenko and co-workers [61] have demonstrated that expression of ATF6α causes diminished CHOP transcription in ER-stressed alveolar epithelial cells. Nonetheless, the ER stress responses are known to be highly redundant and there is extensive cross talking among different pathways. Therefore, none of these genes are exclusively controlled by ATF6α [62], although most of them contain UPRE sequences at their promoter sites.

Up to this point we demonstrated that VACV infections induce UPR through the activation of ATF6α, which in turn may modulate other branches of the ER stress signaling, particularly the IRE1 pathway. By asking whether ATF6α activation plays a role in VACV replication, we investigated this event in ATF6α KO cells and compared with WT cells. When we analyzed VACV-WR growth curves in wild typed MEFs or in ATF6α KO fibroblasts we did not see important differences in virus yield (IMV production, which is the virus particle evaluated is such experiments).On the other hand, virus plaque sizes in the absence of ATF6α were consistently smaller than those seen on WT fibroblasts. Moreover, CPE observed on ATF6α KO cells seemed attenuated when compared to normal cells, and cells deficient in ATF6α presented significantly reduced viral gene expression. Interestingly, genetic complementation of the expression of ATF6α transcription factor in deficient cells was able to boost virus yield as well as to rescue CPE to wild type virus patterns (not shown). One possible explanation to why no differences were seen in virus yield in the growth curve experiments is that primary MEFs were otherwise infected, different from the transformed ATF6α knockout cells. Therefore, the intrinsic differences between these two cells could have masked possible differences in virus yield. Nonetheless, we saw no differences in virus morphogenesis either in the presence or in absence of ATF6α.

Collectively, our results indicate that VACV induces and exploits ATF6α signaling and the UPR upon infection in order to maximize its replicative success. Nonetheless, the data shows that ATF6α and target genes are not required for virus replication, but may represent a viral strategy to boost virus yield in infected cells. Indeed, the exploitation of host signaling pathways by VACV in order to potentiate virus replication has been frequently described [41, 63–65]. Similar trend of UPR activation and regulation has been described for other viruses such as *Myxoma virus* [18], another member of *Poxviridae* family, as well as for other DNA viruses including members of *Herpesviridae* [39] and *Asfaviridae* [40] families. It is also important to mention that the use of UPR by VACV to achieve maximum viral replication is rather selective. We evaluated VACV replication in PERK knockout cells in comparison to PERK WT cells and observed no differences in virus replication parameters, making this branch of the UPR apparently irrelevant for the VACV replicative success (data not shown). Further experimentation, however, will be necessary to thoroughly explore this hypothesis.

## Conclusions

This work reveals how VACV manipulates and interacts in a complex way with cell signaling pathways starting from the ER, showing how the virus-host cell interaction is critical in determining the efficiency of a viral multiplication cycle, and how the absence of proteins with important roles in homeostasis cell, such as ATF6 from the UPR pathway, can interfere with this process.

## Data Availability

Data and materials are available upon request from the corresponding author.

## Abbreviations

AraC: Cytosine arabinoside
ATF6: Activating transcription fator 6
bZIP: Basic leucine zipper
BAX: Bcl-2-associated X protein
BiP: Binding immunoglobulin protein
CPE: Cytophatic effect
CEFs: Chicken embryo fibroblasts
CEV: Cell associated enveloped virus
CHOP: CCAAT-*enhancer-binding protein homologous protein*
D-MEM: Dulbecco's modified eagle medium
DDIT3: DNA damage-inducible transcript 3
DNA: Deoxyribonucleic acid
DEPC: Diethypyrocarbonate
GFP: Green fluorescent protein
IMV: Intracellular mature virus
IRE1: Inositol-requiring enzyme 1
mRNA: Messenger RNA
MVA: Modified vaccinia virus ankara
MEF: Mouse embryonic fibroblasts
M-MLV: Moloney murine leukemia virus
NEAA: Non-essential amino acids
NYCBH: New York City Board of Health
ORF: Open reading frame
PBS: Phosphate buffered saline
PCR: Polymerase chain reaction
PDI: Protein disulfide isomerase
PDIA6: Protein disulfide isomerase-associated 6
PERK: PKR-like endoplasmic reticulum kinase
ER: Endoplasmic reticulum
S1P: Site-1 protease
S2P: Site-2 protease
SFB: Fetal bovine serum
UPR: Unfolded protein response
VACV: Vaccínia virus
VACV WR: Vaccinia virus Western Reserve
VGF: Vaccinia growth factor
XBP1: X-box binding protein 1
sXBP1: Processed form of XBP1
uXBP1: Processed form of XBP1
WT: Wild type

## References

[1] Osborne J, Da Silva M, Am F, Sa S, Olsen-Rasmussen M, Upton C, Rm B, Chen N, Feng Z, Rl R, Liu J, Pougatcheva S, Chen W, Rm W, Jj E (2007). Genomic differences of vaccinia virus clones from Dryvax smallpox vaccine: the Dryvax-like ACAM2000 and the mouse neurovirulent clone-3. Vaccine.

[2] Qin L, Upton C, Hazes B, Evans Dh (2011). Genomic analysis of the vaccinia virus strain variants found in Dryvax vaccine. J Virol.

[3] Zhang Q, Tian M, Feng Y, Zhao K, Xu J, Liu Y, Shao Y (2013). Genomic sequence and virulence of clonal isolates of vaccinia virus Tiantan, the Chinese smallpox vaccine strain. PLoS ONE.

[4] Parker RF, Bronson LH, Green RH (1941). Further studies on the infectious unit of vaccinia. J Exp Med.

[5] Law M, Putz MM, Smith GL (2005). An investigation of the therapeutic value of vaccinia-immune IgG in a mouse pneumonia model. J Gen Virol.

[6] Prazsák I, Tombácz D, Szűcs A, Dénes B, Snyder M, Boldogkői Z (2018). Full genome sequence of the western reserve strain of vaccinia virus determined by third-generation sequencing. Genome Announc.

[7] Carroll MW, Moss B (1997). Host range and cytopathogenicity of the highly attenuated MVA strain of vaccinia virus: propagation and generation of recombinant viruses in a nonhuman mammalian cell line. Virology.

[8] Jl N, Ce G, Domingo-Gil E, Mm G, Esteban M (2006). Cellular and biochemical differences between two attenuated poxvirus vaccine candidates (MVA and NYVAC) and role of the C7L gene. J Virol.

[9] Jc G-G, Risco C, Rodríguez D, Cabezas P, Guerra S, Jl C, Esteban M (2005). Differences in virus-induced cell morphology and in virus maturation between MVA and other strains (WR, Ankara, and NYCBH) of vaccinia virus in infected human cells. J Virol.

[10] Meyer H, Sutter G, Mayr A (1991). Mapping of deletions in the genome of the highly attenuated vaccinia virus MVA and their influence on virulence. J Gen Virol.

[11] Antoine G, Scheiflinger F, Dorner F, Falkner FG (1998). The complete genomic sequence of the modified vaccinia Ankara strain: comparison with other orthopoxviruses. Virology.

[12] Tolonen N, Doglio L, Schleich S, Krijnse LJ (2001). Vaccinia virus DNA replication occurs in endoplasmic reticulum-enclosed cytoplasmic mini-nuclei. Mol Biol Cell.

[13] Fagone P, Jackowski S (2009). Membrane phospholipid synthesis and endoplasmic reticulum function. J Lipid Res.

[14] Walter P, Ron D (2011). The unfolded protein response: from stress pathway to homeostatic regulation. Science.

[15] Zhang L, Wang A (2012). Virus-induced ER stress and the unfolded protein response. Front Plant Sci.

[16] Mei Y, Thompson MD, Cohen RA, Tong X (2011). Endoplasmic reticulum stress and related pathological processes. J Pharmacol Biomed.

[17] Wang X, Lin P, Yin Y, Zhou J, Lei L, Zhou X, Jin Y, Wang A (2015). Brucella suis vaccine strain S2-infected immortalized caprine endometrial epithelial cell lines induce non-apoptotic ER-stress. Cell Stress Chaperones.

[18] Km D, My B, Bartee E (2015). Myxoma virus attenuates expression of activating transcription factor 4 (ATF4) which has implications for the treatment of proteasome inhibitor–resistant multiple myeloma. Oncol Virother.

[19] Ron D, Walter P (2007). Signal integration in the endoplasmic reticulum unfolded protein response. Nat Rev Mol Cell Biol.

[20] Mariciniak SJ, Ron D (2006). Endoplasmic stress signaling in disease. Physiol Rev.

[21] Hollien J (2013). Evolution of the unfolded protein response. Biochim Biophys Acta.

[22] Hernandez R, Brown DT (2010). Growth and maintenance of chick embryo fibroblasts (CEF). Curr Protoc Microbiol.

[23] Cotter CA, Earl PL, Wyatt LS, Moss B (2015). Preparation of cell cultures and vaccinia virus stocks. Curr Protoc Microbiol.

[24] Lee K, Tirasophon W, Shen X, Michalak M, Prywes R, Okada T, Yoshida H, Mori K, Kaufman RJ (2002). IRE1α-mediated unconventional mRNA splicing and S2P-mediated ATF6 cleavage merge to regulate XBP1 in signaling the unfolded protein response. Genes Dev.

[25] Wk J (1962). The purification of four strains of poxvirus. Virology.

[26] Mas C, Eg K (1993). Critical period for irreversible block of vaccinia virus replication. Rev Microbiol.

[27] Wang Y, Shen J, Arenzana N, Tirasophon W, Kaufman RJ, Prywes R (2000). Activation of ATF6 and an ATF6 DNA binding site by the endoplasmic reticulum stress response. J Biol Chem.

[28] Nadanaka S, Yoshida H, Kano F, Murata M, Mori K (2004). Activation of mammalian unfolded protein response is compatible with the quality control system operating in the endoplasmic reticulum. Mol Biol Cell.

[29] Calfon M, Zeng H, Urano F, Till JH, Hubbard SR, Harding HP, Clark SG, Ron D (2002). IRE1 couples endoplasmic reticulum load to secretory capacityby processing the XBP-1 mRNA. Nature.

[30] Vandesompele J, De Preter K, Pattyn F, Poppe B, Van Roy N, De Paepe A, Speleman F (2002). Accurate normalization of real-time quantitative RT-PCR data by geometric averaging of multiple internal control genes. Genome Biol.

[31] Mügge FLB, Silva AM (2017). Aspirin metabolite sodium salicylate selectively inhibits transcriptional activity of ATF6α and downstream target genes. Sci Rep.

[32] Shen J, Snapp EL, Lippincott-Schwartz J, Prywes R (2005). Stable binding of ATF6 to BiP in the endoplasmic reticulum stress response. Mol Cell Biol.

[33] Costa GB, Borges IA, Alves PA, Miranda JB, Luiz AP, Ferreira PC, Abrahão JS, Moreno EC (2015). Alternative Routes of Zoonotic Vaccinia Virus Transmission. Brazil. Emerg Infect Dis..

[34] Leão TL, Da Fonseca FG (2014). Subversion of cellular stress responses by poxviruses. World J Clin Infect Dis.

[35] Mori K (2009). Signaling pathways in the unfolded protein response: development from yeast to mammals. J Biochem.

[36] Nishitoh H (2012). CHOP is a multifunctional transcription factor in the ER stress response. J Biochem.

[37] Liou HC, Boothby MR, Finn PW, Davidon R, Nabavi N, Zeleznik-Le NJ, Ting JP, Glimcher LH (1990). A new member of the leucine zipper class of proteins that binds to the HLA DR alpha promoter. Science.

[38] Yoshida H, Matsui T, Yamamoto A, Okada T, Mori K (2001). XBP1 mRNA is induced by ATF6 and spliced by IRE1α in response to ER stress to produce a highly active transcription factor. Cell.

[39] Burnett HF, Audas TE, Liang G, Lu RR (2012). Herpes simplex virus-1 disarms the unfolded protein response in the early stages of infection. Cell Stress Chaperones.

[40] Galindo I, Hernáez B, Muñoz-Moreno R, Cuesta-Geijo MA, Dalmau-Mena I, Alonso C (2012). The ATF6 branch of unfolded protein response and apoptosis are activated to promote African swine fever virus infection. Cell Death Dis.

[41] Pechenick Jowers T, Featherstone RJ, Reynolds DK, Brown HK, James J, Prescott A, Haga IR, Beard PM (2015). RAB1A promotes vaccinia virus replication by facilitating the production of intracellular enveloped virions. Virology.

[42] Moss B (2015). Poxvirus membrane biogenesis. Virology.

[43] Maruri-Avidal L, Weisberg AS, Bisht H, Moss B (2013). Analysis of viral membranes formed in cells infected by a vaccinia virus L2-deletion mutant suggests their origin from the endoplasmic reticulum. J Virol.

[44] Weisberg AS, Maruri-Avidal L, Bisht H, Hansen BT, Schwartz CL, Fischer ER, Meng X, Xiang Y, Moss B (2017). Enigmatic origin of the poxvirus membrane from the endoplasmic reticulum shown by 3D imaging of vaccinia virus assembly mutants. Proc Natl Acad Sci U S A.

[45] Pathak PK, Peng S, Meng X, Han Y, Zhang B, Zhang F, Xiang Y, Deng J (2018). Structure of a lipid-bound viral membrane assembly protein reveals a modality for enclosing the lipid bilayer. Proc Natl Acad Sci U S A.

[46] Pincus D, Chevalier MW, Aragón T, Van Anken E, Se V, El-Samad H, Walter P (2010). BiP binding to the ER-stress sensor IRE1α tunes the homeostatic behavior of the unfolded protein response. PLoS Biol.

[47] Lisbona F, Rojas-Rivera D, Thielen P, Zamorano S, Todd D, Martinon F, Glavic A, Kress C, Lin JH, Walter P, Reed JC, Glimcher LH, Hetz C (2009). BAX Inhibitor-1 is a negative regulator of the ER stress sensor IRE1α. Mol Cell.

[48] Korennykh AV, Egea PF, Korostelev AA, Finer-Moore J, Stroud RM, Zhang C, Shokat KM, Walter P (2011). Cofactor-mediated conformational control in the bifunctional kinase/RNase IRE1α. BMC Biol.

[49] Korennykh A, Korostelev AA, Egea PF, Finer-Moore J, Stroud RM, Zhang C, Shokat KM, Walter P (2011). Structural and functional basis for RNA cleavage by IRE1α. BMC Biol.

[50] Hetz C (2013). The biological meaning of the UPR. Nat Rev Mol Cell Biol.

[51] Walter F (2018). ER stress signaling has an activating transcription factor 6α (ATF6)-dependent "off-switch". J Biol Chem.

[52] Martinon F, Chen X, Lee A, Glimcher L (2010). TLR activation of the transcription factor XBP1 regulates innate immune responses in macrophages. Nat Immunol.

[53] Dong H (2019). The IRE1 endoplasmic reticulum stress sensor activates natural killer cell immunity in part by regulating c-Myc. Nat Immunol.

[54] Kamimura D, Bevan MJ (2008). Endoplasmic reticulum stress regulator XBP-1 contributes to effector CD8+ T cell differentiation during acute infection. J Immunol.

[55] Roach JC, Smith KD, Strobe KL, Nissen SM, Haudenschild CD, Zhou D, Vasicek TJ, Held GA, Stolovitzky GA, Hood LE, Aderem A (2007). Transcription factor expression in lipopolysaccharide-activated peripheral-blood-derived mononuclear cells. Proc Natl Acad Sci USA.

[56] Richardson CE, Kooistra T, Kim DH (2010). An essential role for XBP-1 in host protection against immune activation in C elegans. Nature.

[57] Vekich JA, Belmont PJ, Thuerauf DJ, Glembotski CC (2012). Protein disulfide isomerase-associated 6 is an ATF6-inducible ER stress response protein that protects cardiac myocytes from ischemia/reperfusion-mediated cell death. J Mol Cell Cardiol.

[58] Eletto D, Eletto D, Dersh D, Gidalevitz T, Argon Y (2014). Protein disulfide isomerase A6 controls the decay of IRE1α signaling via disulfide-dependent association. Mol Cell.

[59] Beck SE, Rodriguez RA, Hawkins MA, Hargy TM, Tc L, Linden KG (2016). Comparison of UV-induced inactivation and RNA damage in MS2 phage across the germicidal UV spectrum. Appl Environ Microbiol.

[60] Yakimovich A (2017). Inhibition of poxvirus gene expression and genome replication by bisbenzimide derivatives. J Virol.

[61] Klymenko O, Huehn M, Wilhelm J, Wasnick R, Shalashova I, Ruppert C, Henneke I, Hezel S, Guenther K, Mahavadi P, Samakovlis C, Seeger W, Guenther A (2019). Regulation and role of the ER stress transcription factor CHOP in alveolar epithelial type-II cells. J Mol Med.

[62] Takayanagi S, Fukuda R, Takeuchi Y, Tsukada S, Yoshida K (2012). Gene regulatory network of unfolded protein response genes in endoplasmic reticulum stress. Cell Stress Chaperones.

[63] Haga IR, Pechenick Jowers T, Griffiths SJ, Haas J, Beard PM (2014). TRAF2 facilitates vaccinia virus replication by promoting rapid virus entry. J Virol.

[64] Harrison K (2016). Vaccinia virus uses retromer-independent cellular retrograde transport pathways to facilitate the wrapping of intracellular mature virions during virus morphogenesis. J Virol.

[65] Bonjardim CA (2017). Viral exploitation of the MEK/ERK pathway—a tale of vaccinia virus and other viruses. Virology.

